# Load transmission via the supraspinatus cord prevents muscle fatty degeneration, a biomechanical study

**DOI:** 10.1016/j.jseint.2024.12.024

**Published:** 2025-02-19

**Authors:** Omar E. Rodriguez-Alejandro, Bethany G. Ricci, Christopher C. Schmidt, Sean P. Cooke, Austin J. Cook, Justin G. Buce, Joshua D. Dworkin, Mark C. Miller, Patrick J. Smolinski

**Affiliations:** a Shoulder and Elbow Mechanical Research Laboratory, Department of Orthopaedic Surgery, University of Pittsburgh Medical Center, Pittsburgh, PA, USA; b Department of Orthopaedic Surgery, Sports Medicine, Cleveland Clinic, Cleveland, OH, USA; c Department of Orthopaedic Surgery, University of Pittsburgh Medical Center, Pittsburgh, PA, USA; d Department of Mechanical Engineering and Material Science, University of Pittsburgh, Pittsburgh, PA, USA; e University of Illinois, College of Medicine, Chicago, IL, USA; f Benton Franklin Orthopedic Associates, Kennewick, WA, USA

**Keywords:** Coracohumeral ligament, Rotator cuff tears, Supraspinatus cord, Fatty degeneration, Abduction force, Rotator cuff force transmission

## Abstract

**Background:**

Location of small- to medium-sized (<30 mm) rotator cuff tears can predict (supraspinatus [SS]) muscle fatty degeneration. It has been hypothesized that SS fatty degeneration occurs because of loss of force transfer by either the coracohumeral ligament (CHL) or the SS cord. The CHL is the anterior insertion of the rotator cable theorized to carry rotator muscle force to the humerus. In this study, we aimed to map the anatomic insertions of the CHL and superior rotator cuff onto the humeral head and then sequentially release the footprint to determine which structures are the most critical for force transmission.

**Methods:**

Twenty fresh-frozen cadaveric specimens (average age 69 ± 10 years, 9 males) were tested in a shoulder simulator under physiological conditions at 0° and 30° of shoulder abduction. After cyclic loading, shoulder abduction force and glenohumeral translation were measured for the native condition and after each humeral footprint release. Ten specimens were assigned randomly to a CHL-first footprint release with sequential release of the SS cord, SS strap, and infraspinatus tendons. The other 10 specimens underwent an SS cord-first footprint release, with sequential release of remaining insertions. Following mechanical testing, soft tissue cross-sectional dimensions and footprint widths, lengths, and areas were scanned for three-dimensional modeling and dimensionally quantified.

**Results:**

An SS cord-first release decreased abduction force by 10% at 0° of abduction and 22% at 30° (*P* = .047); further, releasing the CHL did not influence the abduction force values (*P* ≥ .091). A CHL first-release or a CHL release after an SS cord release did not result in a decrease in abduction force (*P* ≥ .081). However, the abduction force with a subsequent SS cord release did significantly decrease (*P* ≤ .047). The average anterior-to-posterior humeral footprint widths of the CHL, SS cord, SS strap, and infraspinatus tendons were 0 mm-3 mm, 3 mm-11 mm, 11 mm-20 mm, and 20 mm-40 mm behind the bicipital groove, respectively.

**Conclusion:**

The SS cord, and not the CHL, is the key structure responsible for the transmission of anterior shoulder abduction force. Relating the clinical tear location with the study’s humeral footprint results indicates the SS cord is intact in small- to medium-sized rotator cuff tears without SS fatty degeneration. Repairing the SS cord in small to medium rotator cuff tears could be efficacious in improving abduction strength and preventing SS muscle fatty degeneration.

Previous investigators have shown that tear location for small- to medium-sized (<30 mm) rotator cuff tears can presage the occurrence of supraspinatus (SS) muscle fatty degeneration.^18^^,^^24^ Using clinical ultrasound (US) examinations, small to medium anterior rotator cuff tears were grouped into intact SS anterior tendon (Fig. 1, *A*) and disrupted SS anterior tendon (Fig. 1, *B*).^24^ In the intact anterior tendon group, the SS tear started on average 10 ± 4 mm behind the bicipital tendon/groove and progressed in a posterior direction 11 ± 4 mm, while the disrupted anterior group’s SS tear initiated on average 0 ± 1 mm from the bicipital tendon/groove and propagated 20 ± 7 mm posterior. The torn SS anterior tendon group vs. the intact group demonstrated a significantly (*P* < .0001) greater amount of SS muscle fatty degeneration.^24^ The investigators hypothesized that fatty degeneration did not ensue in the intact group because the anterior SS tendon maintained SS muscle force transfer to the humerus.^18^^,^^24^ US studies were essential in grouping small- to medium-sized rotator cuff tears into either intact or disrupted anterior tendon.^24^ However, US studies are not precise enough to identify the key anatomic structure or structures believed to be responsible for preventing the SS muscle deterioration.^24^

**Figure 1:**
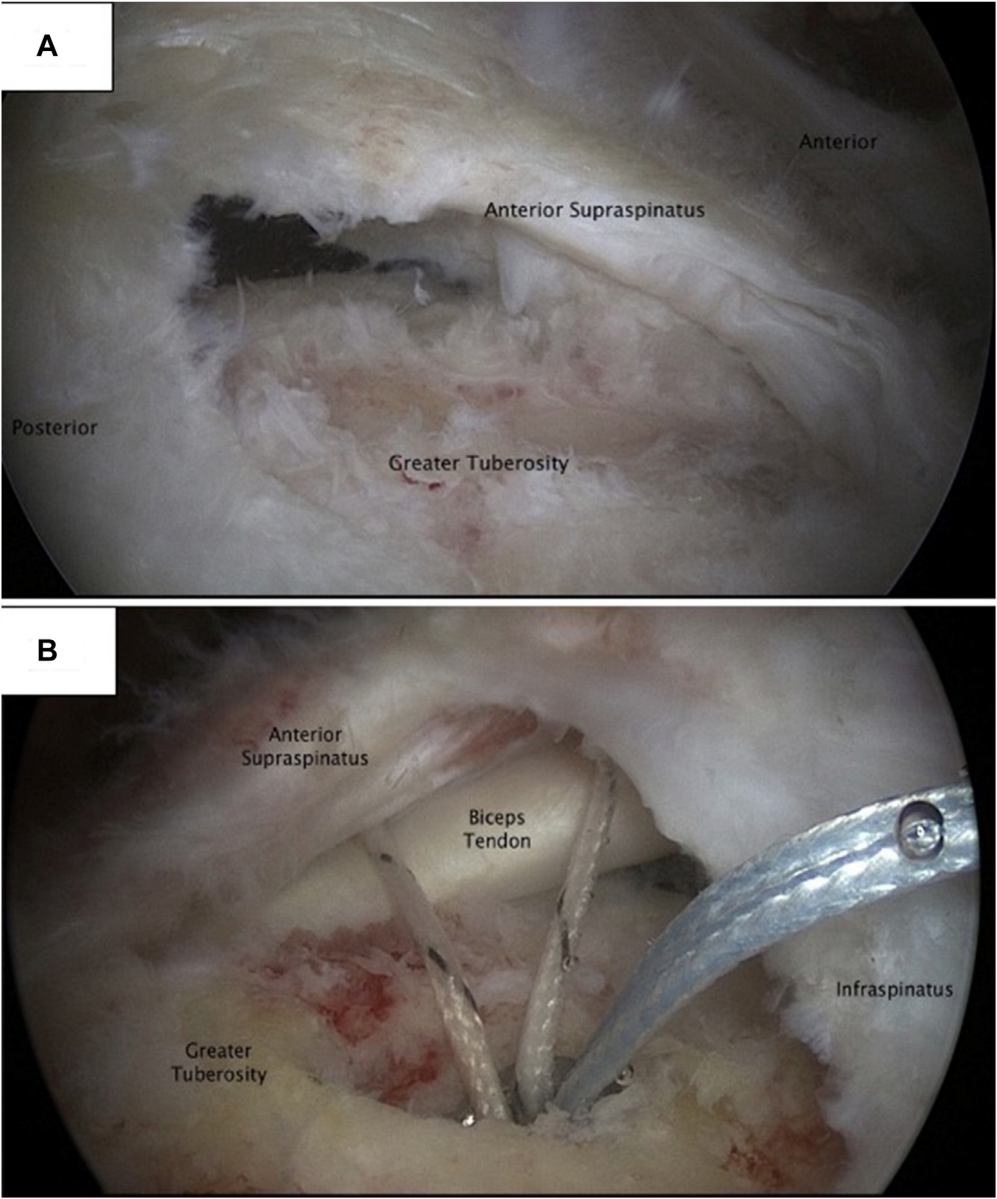
(**A**) An arthroscopic picture showing the anterior tendon is intact. Notice the biceps tendon cannot be visualized. (**B**) An arthroscopic picture depicting a torn anterior tendon. Notice the biceps tendon is easily seen.

Reprinted with modifications from the Journal of Shoulder and Elbow Surgery, 23(1), Namdari S, et al.,^24^ with permission from Elsevier.

The coracohumeral ligament (CHL) and the SS tendon are the only structures located immediately posterior to the bicipital groove, and thus, these structures may be responsible for preventing SS fatty infiltration as seen in the intact SS anterior tendon group.^7^^,^^24^ The rotator cable is a deep semilunar band of thin collagen fibers that surrounds the lateral SS and infraspinatus (IS) tendons that does not have its own humeral footprint because its anterior fibers blend into the deep fibers of the CHL.^6^^,^^7^ In other words, the anterior rotator cable inserts into the anterior humerus through the CHL.^6^^,^^7^ The CHL lies over the long head of the bicipital tendon and its groove; its humeral footprint is positioned between the subscapularis (SSc) and SS tendon (Fig. 2, *A*).^7^ Immediately adjacent to the CHL is the SS tendon, which can be divided into two anatomic subunits: anterior SS cord and posterior SS strap (Fig. 2, *B*). ^7^^,^^11^^,^^25^^,^^30^^,^^34^ The muscular cross-sectional area of the SS cord is 2.3 times larger than the SS strap (*P* < .001), implying that the SS cord can generate more contractile force than the SS strap.^30^^,^^34^

**Figure 2:**
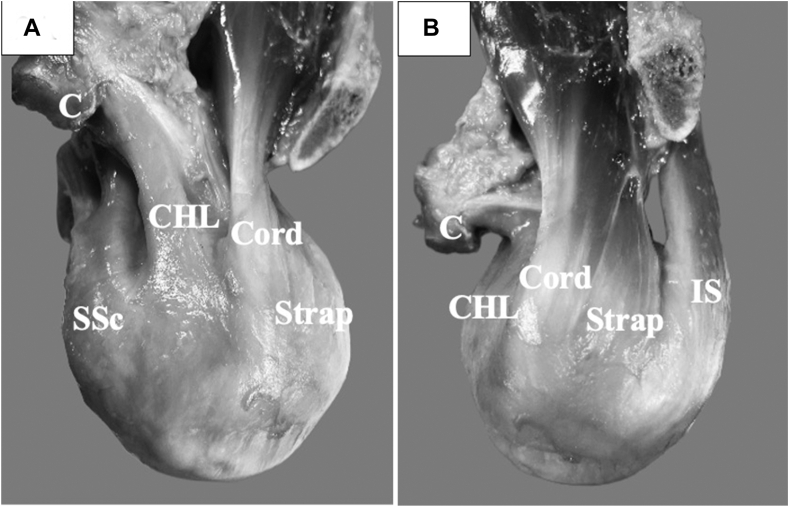
(**A**) A photograph of a specimen showing the coracohumeral ligament (CHL) sandwiched between the subscapularis muscle (SSc) and the supraspinatus (SS) cord (Cord), coracoid (C), SS strap (Strap), and infraspinatus (IS). (**B**) A photograph illustrating the relationship between the SS cord (Cord) and SS strap (Strap). The connection between the CHL and the rotator cable cannot be visualized in these pictures because it is an articular-sided structure.

(B) Reprinted from the *Journal of Shoulder and Elbow Surgery*, 33(1), Schmidt CC, et al.,^31^ with permission from Elsevier.

The CHL is a ligamentous structure and is not expected to transfer active rotator cuff load to the humerus. The CHL, SS cord, and SS strap are discrete structures with separate humeral footprints, but their collagen fibers do interdigitate into each other.^7^^,^^15^^,^^16^^,^^30^ This interdigitation may dynamize the CHL and expand the functional footprint of the SS cord to include parts of the CHL footprint. Further, the rotator cable had been thought to carry SS and IS muscular contractile forces to the anterior humerus through the CHL.^6^^,^^13^^,^^21^ Currently, conflicting data exist whether the CHL’s humeral footprint receives abduction load from the rotator cable.^13^^,^^21^^,^^32^^,^^35^^,^^36^

To our knowledge, there have been no biomechanical studies comparing shoulder abduction force transmission of the CHL to the SS cord. Despite the structural interdigitations of the CHL and the SS cord, their role in rotator cuff force transfer may be different. In theory, an SS tear with a disrupted anterior tendon may reduce SS force transmission enough to cause the observed SS fatty degeneration.^24^ SS fatty degeneration can be multifactorial, but loss of rotator cuff tension has been reported to cause irreversible muscle atrophy, fatty infiltration, and fibrosis.^12^

The goal of this study is to understand the structural and insertional anatomy of the CHL, SS cord, and SS strap tendons and determine the relative contributions of the CHL and SS cord to shoulder abduction force. We hypothesized that both the CHL and the SS cord are the main structures injured in SS tendon tears with disruption of the anterior tendon, but the SS cord tendon plays a greater role in force transmission during shoulder abduction.

## Materials and methods

### Experimental design

This is a biomechanical study investigating the CHL and SS cord structures and their role in shoulder abduction force transfer.

**Figure 3:**
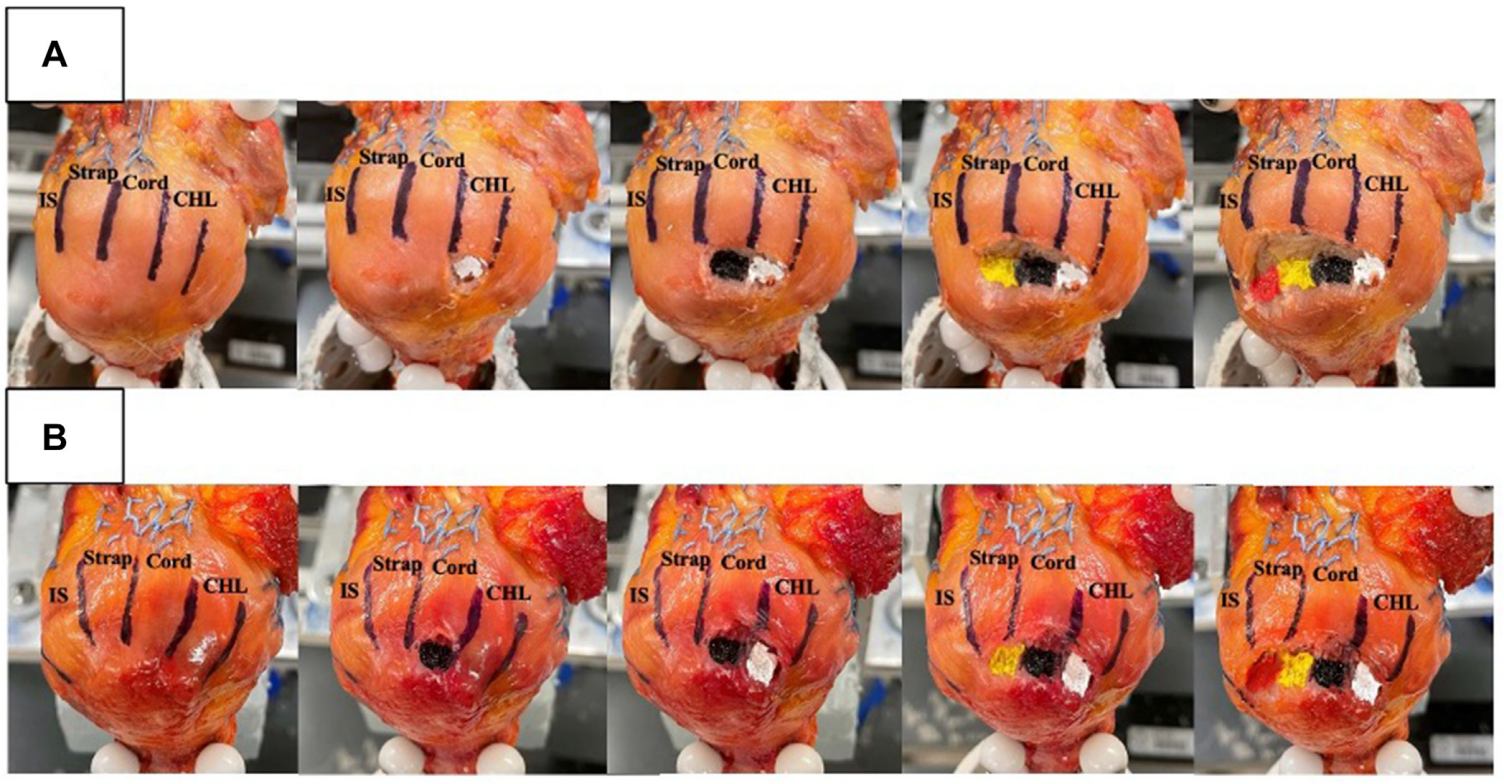
(**A**) A series of photographs demonstrating a coracohumeral (CHL)-first release. Notice that after each release, the respective footprints were painted with consistent colors: CHL white, supraspinatus (SS) cord (cord) black, SS strap (strap) yellow, infraspinatus (IS) red. (**B**) The sequence for an SS cord-first release group.

Twenty fresh-frozen human cadaveric specimens were secured into a validated shoulder simulator, and all the rotator cuff muscles, including the SS cord and SS strap tendons, were loaded with physiological forces at 0° and 30° of abduction.^32^ Mechanical variables measured were shoulder abduction force and glenohumeral translation. After cyclic loading of the shoulder and baseline testing, 10 specimens were assigned randomly to a CHL-first footprint release with sequential release of the SS cord, SS strap, and IS tendons (Fig. 3, *A*). The other 10 specimens underwent an SS cord-first footprint release, with sequential release of remaining rotator cuff tendons (Fig. 3, *B*). The underlying capsule was also released at each respective tendon release, and a freer elevator was used to confirm complete capsular release. After each structural release, the respected footprint was painted for later anatomic analysis: CHL-white, SS cord-black, SS strap-yellow, and IS-red. Only the first release of either the CHL-first or SS-first cord footprint was randomized. Mechanical testing of each specimen was performed after each footprint release. After mechanical testing, soft tissue cross-sectional dimensions and footprint width, lengths, and areas were quantified.

#### Specimen preparation

With institutional approval, 45 fresh-frozen cadaveric arms from scapula to fingertip were obtained for this study. Twenty-five specimens were excluded because of glenohumeral arthritis and/or rotator cuff pathology. Twenty specimens, including 11 females with an average age of 69 ± 11 years, met the inclusion criteria. The specimen number was based on an a priori sample size calculation (see *Statistical Analysis* section). The specimens were dissected down to the level of the rotator cuff capsular structures. A claviculectomy and an acromionectomy were performed to see the bursal side of the CHL and rotator cuff muscles and tendons. For each specimen, the humerus was then transected 5 cm distal to the deltoid insertion. The anterior and posterior borders of the CHL and rotator cuff were identified under loupe magnification and outlined with a waterproof marker. The SS muscle was dissected into its cord and strap subunits. A medial-lateral line separating the SS cord and SS strap was made based on the muscle anatomy and tendon shape; the SS cord is cylindrical while the SS strap is rectangular.^29^ A locking #2 braided suture (Fiberwire; Arthrex, Naples, FL, USA) was sewn into the myotendinous junction of the SS cord, SS strap, IS, teres minor, and upper and lower SSc. Eyelet screws were fixed into the scapula along the anatomic lines of muscle pull. The scapula and humerus of each specimen were fixed into a custom-built fixture, scapular box, using bolts and polyester resin (Bondo; 3M, St. Paul, MN, USA). The scapular box was secured to the shoulder simulator. Using low-friction cables, each of the six myotendinous sutures was connected to their respective individual actuators within the shoulder simulator. Eight white spherical markers (four on the humerus and four on the scapula) were solidly screwed into the bone of each specimen before mechanical testing and were used to track motion between the scapula and humerus.

##### Shoulder simulator, mechanical testing, and humeral head translation

**Figure 4:**
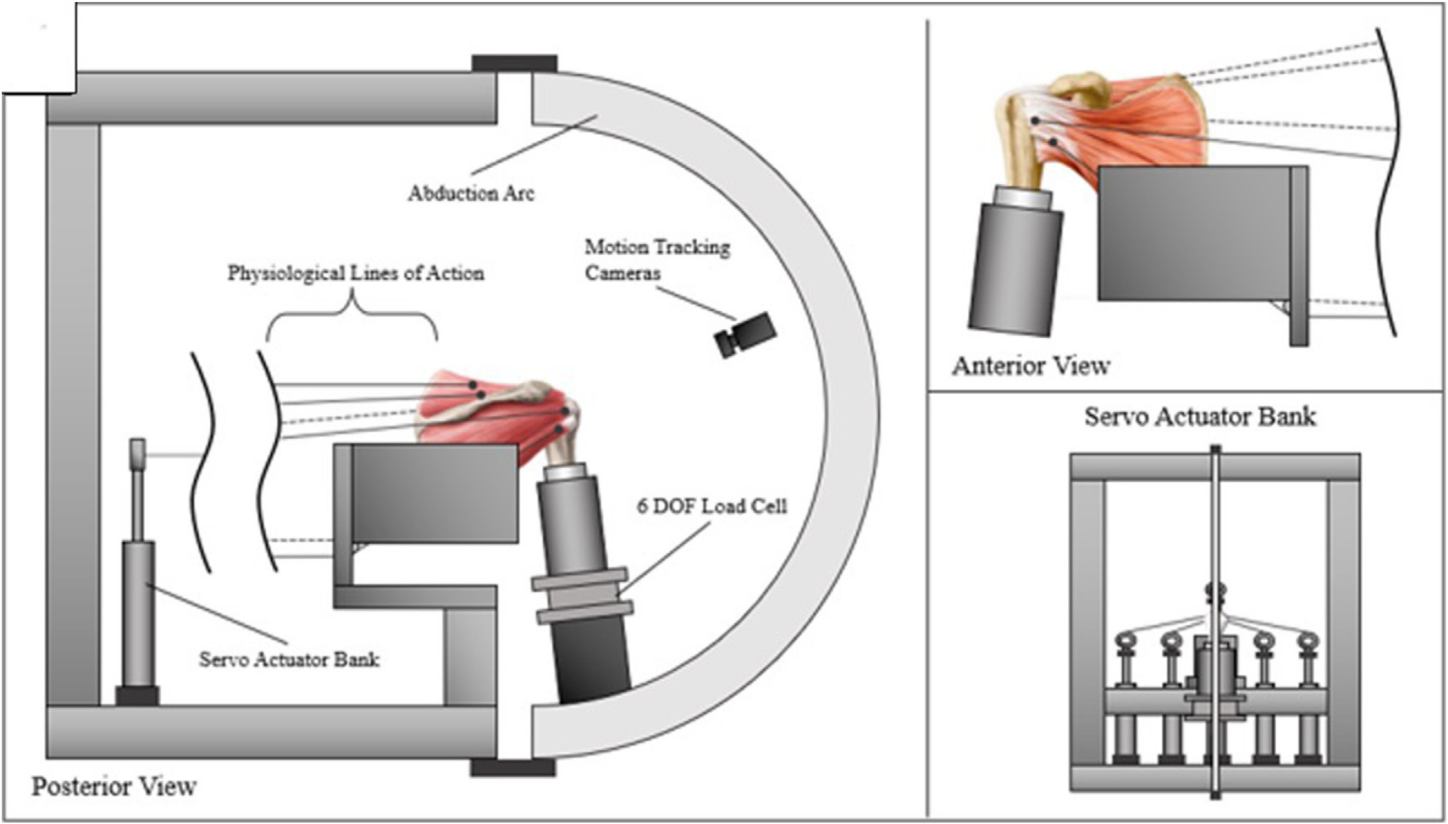
A schematic of the validated shoulder simulator.

The shoulder simulator provided physiological loading conditions to each tied muscle using 6 servo-driven actuators (Parker Hannifin, Cleveland, OH, USA) fixed to a metal frame (Fig. 4).^32^ The details of the shoulder simulator, mechanical testing, and humeral head translation have been previously published, and the details are included in a Supplementary Methods.^1^^,^^17^,^19^,^20^,^28^^,^^31^, ^32^, ^33^

*DOF*, degree-of-freedom. Adapted with permission from Wolters Kluwer Health, Inc.: [Schmidt CC, Spicer CS, Papadopoulos DV, Delserro SM, Tomizuka Y, Zink TR, et al., *The rotator cable does not stress shield the crescent area during shoulder abduction.*, *The Journal of Bone and Joint Surgery*, 104, 14, 1292-1300. https://journals.lww.com/jbjsjournal/fulltext/2022/07200/the_rotator_cable_does_not_stress_shield_the.9.aspx].

##### Ligament/tendon thicknesses and footprint locations, sizes, and area measurements

**Figure 5:**
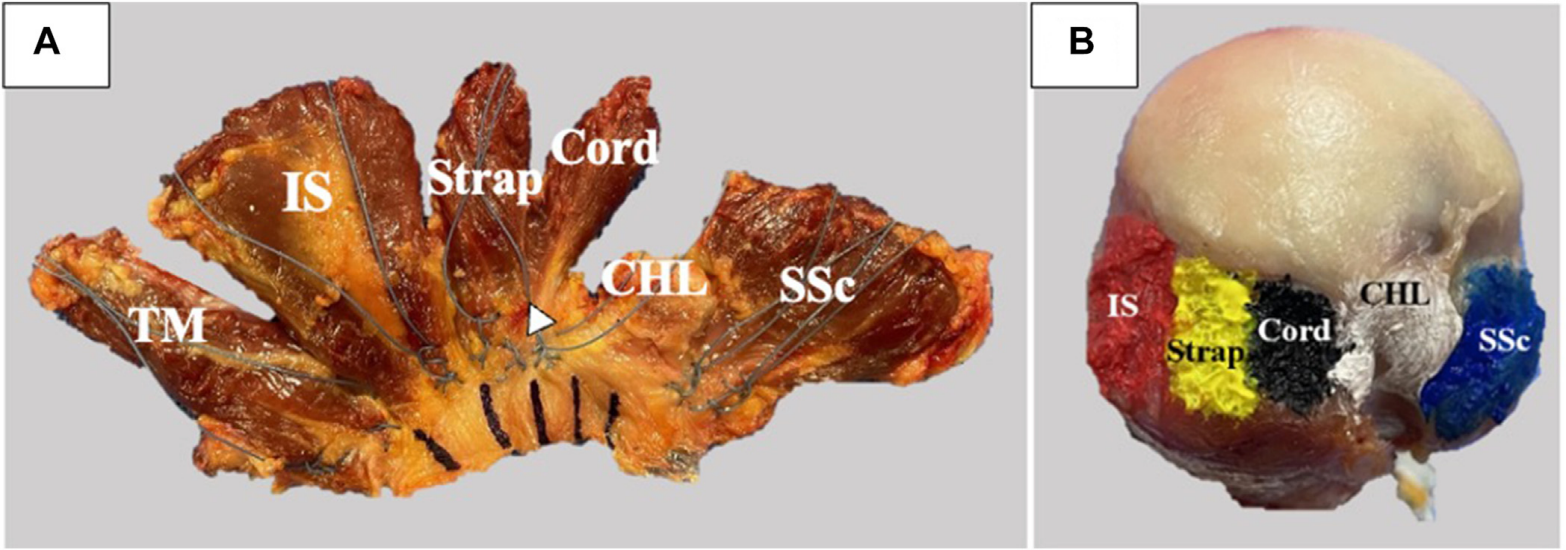
(**A**) Photograph of a dissected rotator cuff capsular complex. Teres minor (TM), infraspinatus (IS), supraspinatus (SS) strap (strap), SS cord (cord), coracohumeral ligament (CHL), and subscapularis (SSc). The arrowhead points to the interconnection between the SS cord and SS strap muscles. (**B**) Picture of dissected humeral footprints. Infraspinatus (IS), SS strap (strap), SS cord (cord), coracohumeral ligament (CHL), and subscapularis (SSc).

**Figure 6:**
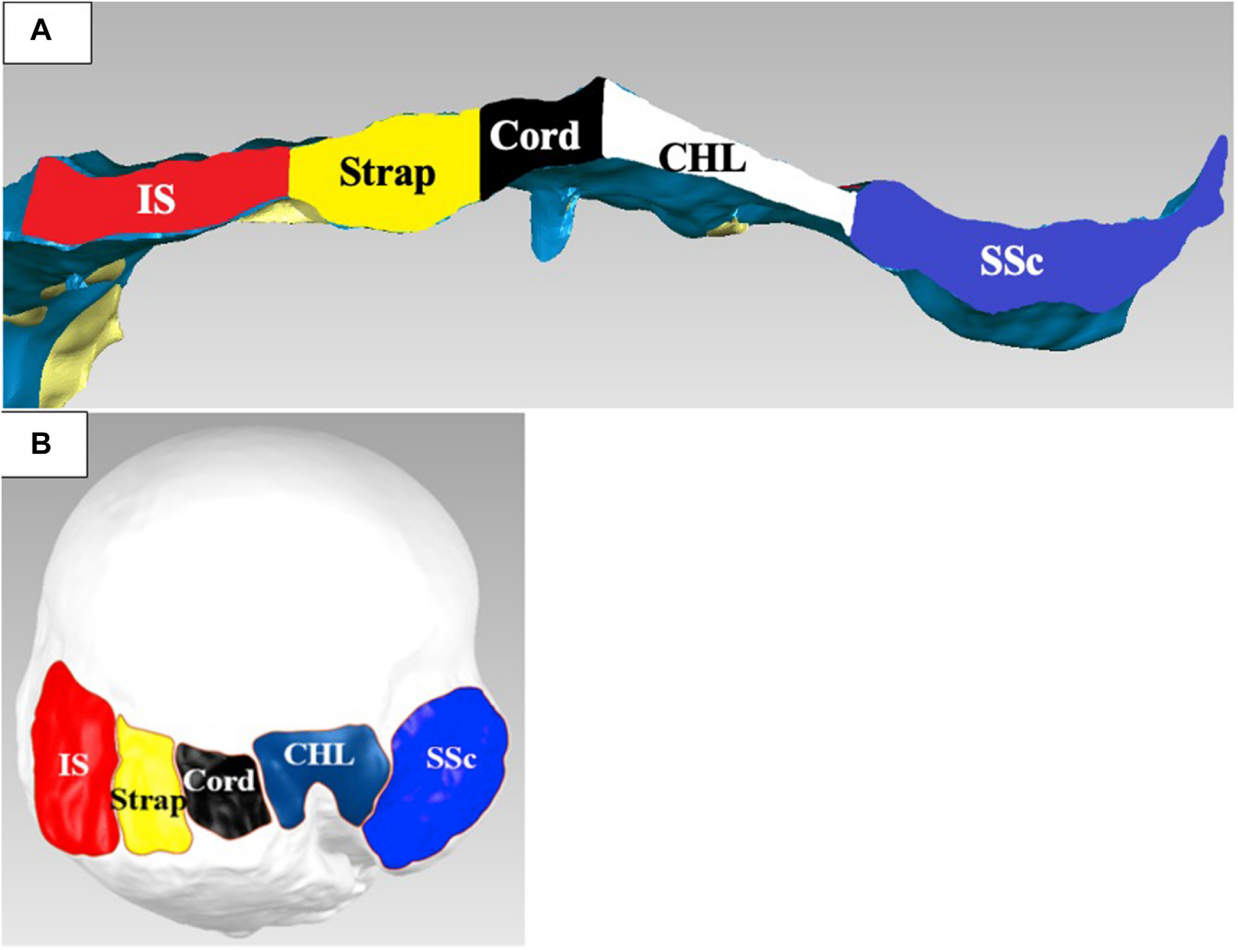
(**A**) Three-dimensional (3D) model of the cross-section of the rotator cuff capsular complex at half the distance from the medial footprint to the musculotendinous junction. Infraspinatus (IS), supraspinatus (SS) strap (strap), SS cord (cord), coracohumeral ligament (CHL), and subscapularis (SSc). (**B**) 3D model of the humeral footprints. Infraspinatus (IS), SS strap (strap), SS cord (cord), coracohumeral ligament (CHL), and subscapularis (SSc).

After mechanical testing, the rotator cuff tendinous capsular complex was sharply separated from the scapula and humerus, leaving the soft tissues (Fig. 5, *A*) and the humeral footprints for measurements (Fig. 5, *B*). The matching 20 rotator cuff tendinous complexes and humeri were laser scanned using a noncontact optical micrometer (FaroArm; Faro Inc., Lake Mary, FL, USA).^32^ Three-dimensional solid replicas of the rotator cuff tendinous complexes and humeri were created from the laser scan data using modeling software (Geomagic; 3D Systems, Rock Hill, SC, USA).^32^ The CHL, SS cord, SS strap, IS thickness (superoinferior), width (anteroposterior), and cross-sectional areas were measured half the distance from the medial footprint to the musculotendinous junction (Fig. 6, *A*). The CHL, SS cord, SS strap, IS insertional footprint locations, anterior-to-posterior widths, medial-to-lateral lengths, and areas were quantified from the models (Fig. 6, *B*). The 20 anatomic measurements were then averaged and reported to the nearest whole millimeter. Our laboratory has previously reported the accuracy of the laser micrometer to be 0.01 mm.^37^

### Statistical analysis

A priori sample size analysis was completed by using previously published mechanical data on increased rotator cuff force requirements for stable humeral abduction between 6 cm and 8 cm tears.^14^ G∗Power (Heinrich Heine Universität, Düsseldorf, Germany) determined that a sample size of 9 was needed to achieve a statistical power of 0.8 and *P* = .05.^31^

Shoulder abduction force was analyzed using a two-factor analysis of variance (ANOVA) for each of the randomized and nonrandomized groups (SPSS; IBM Corp, Armonk, NY, USA). The two factors were humeral release and abduction angle. Significant differences for the randomized portions of the study were compared post hoc with a paired t-test. After releasing the CHL and SS cord, the study was nonrandomized (ie, with the two groups with the first cuts combined), and Bonferroni post-hoc pairwise comparisons were used to compare the subsequent release of the SS strap and IS to the previous release cases and each other. Humeral translation was analyzed using a three-factor ANOVA. The three factors were humeral release, abduction angle, and direction. Paired t-tests were used to determine significance between anatomic measurements. Significance was set at *P* < .05.

## Results

The shoulder abduction force results comparing the randomized CHL-first and SS cord-first release groups to the native cases are shown in Table I. The two-factor ANOVA showed a significant difference between the two release states (*P* = .016), but not between the two angles of abduction (*P* = .192). In the CHL-first release group, shoulder abduction force decreased to 3.0% at 0° and 7.0% at 30° compared to native; however, this decline was not significant (*P* ≥ .356). Subsequent SS cord release significantly decreased the abduction force to 20% at 0° and 18% at 30° of native (*P* ≤ .045). Releasing the SS cord first significantly decreased the abduction force to 10% at 0° and 22% at 30° (*P* = .047). An additional release of the CHL did not further impact the abduction force when compared to the previous release (*P* ≥ .091). The result shows that the SS cord, and not the CHL, plays an important role in conveying shoulder abduction force.

**Table I:** Abduction forces of the randomized released structures; mean (std).

Abduction angle	Released structure	Abduction force [N]	Percentage of native force	*P* value (vs. native)
CHL-first group				
0°	CHL	5.9 (1.7)	97%	.610
	CHL + SS cord	4.9 (2.0)	80%	.022
30°	CHL	6.7 (3.1)	93%	.356
	CHL + SS cord	5.9 (2.5)	82%	.045
SS cord-first group				
0°	SS cord	4.5 (1.4)	90%	.047
	SS cord + CHL	4.1 (1.5)	82%	.013
30°	SS cord	4.6 (2.0)	78%	.047
	SS cord + CHL	4.3 (1.8)	73%	.012

*CHL*, coracohumeral ligament; *SS*, supraspinatus; *std*, standard deviation

After the release of the CHL and SS cord from their footprints, the subsequent nonrandom rotator cuff releases were significant (*P* < .001) compared to the native state, but no difference was seen in abduction angle (*P* = .445). Table II depicts the abduction values after additional releases of the SS strap and IS tendons off their footprints. In specimens with a CHL and SS cord release, an additional SS strap release resulted in no significant change in abduction force (*P* ≥ .264). However, releasing both the SS strap and IS tendons led to a significant decrease in abduction force of 44% at 0° and 49% at 30° compared to the prior released structure (*P* < .001). The data demonstrate that SS strap and the IS tendons are important in transferring shoulder abduction force.

**Table II:** Abduction forces of nonrandomized released structures; mean (std).

Abduction angle	Released structure	Abduction force [N]	Percentage of native force	*P* value (vs. prior released structure)
0°	SS strap	4.3 (1.3)	77%	>.999
	SS strap + IS	3.7 (1.3)	66%	<.001
30°	SS strap	4.8 (2.3)	73%	.264
	SS strap + IS	3.3 (1.9)	51%	<.001

*SS*, supraspinatus; *IS*, infraspinatus; *std*, standard deviation

Figure 6, *A* illustrates the cross-section of the SSc, CHL, SS cord, SS strap, and IS tendons at their midpoint, half the distance from the medial footprint to their musculotendinous junction. Table III shows the widths, thicknesses, and areas of each of the above structures. The cross-sectional areas of the CHL, SS cord, and SS strap tendons were similar (*P* ≥ .159).

**Table III:** Measurements of the rotator cuff and coracohumeral ligament at the midpoint; mean (std).

Structure	Anteroposterior width (mm)	Thickness (mm)	Cross-sectional area (mm^2^)
CHL	18 (7)	3 (2)	59 (39)
SS cord	7 (3)	6 (2)	54 (24)
SS strap	17 (12)	5 (2)	62 (28)
IS	16 (6)	5 (2)	82 (36)

*SS*, supraspinatus; *IS*, infraspinatus; *CHL*, coracohumeral ligament; *std*, standard deviation

Figure 6, *B* depicts the footprints of the CHL, SS cord, SS strap, and IS tendons. Table IV depicts the humeral footprint widths, lengths, and areas for the above structures. The SS strap footprint area of 77 ± 19 mm^2^ was larger than the SS cord footprint area of 62 ± 20 mm^2^ (*P* = .001). The humeral insertion of the SS cord and SS strap footprints started 3 ± 1 mm and 11 ± 2 mm posterior to the posterior edge of the humeral bicipital groove, respectively.

**Table IV:** Footprint measurements of the rotator cuff and coracohumeral ligament; mean (std).

Structure	Anteroposterior width (mm)	Mediolateral length (mm)	Area (mm^2^)
CHL	12 (2)	10 (2)	89 (30)
SS cord	8 (1)	8 (2)	62 (20)
SS strap	9 (2)	9 (1)	77 (19)
IS	20 (4)	9 (1)	191 (73)

A/P distance from the posterior bicipital groove (mm)	
Anterior SS cord	3 (1)
Posterior SS cord	11 (2)

*SS*, supraspinatus; *IS*, infraspinatus; *CHL*, coracohumeral ligament; *std*, standard deviation

The AP and SI humeral apex motion was <2 mm in all cases, and the ANOVA did not show significance for the release sequence (*P* ≥ .610) or direction (*P* ≥ .795).

## Discussion

The SS cord, and not the CHL, is the key structure responsible for the transmission of anterior shoulder abduction force. A SS cord-first release decreased abduction force to 10% at 0° and 22% at 30° (*P* = .047); further, releasing the CHL did not influence the abduction force values (*P* ≥ .091). A CHL-first release or a CHL release after an SS cord release did not result in a decrease in abduction force (*P* ≥ .081). However, the abduction force did significantly decrease 11% and 22% with a secondary SS cord release (*P* ≤ .047).

SS muscle fatty degeneration occurs more frequently in small- to medium-sized (<30 mm) SS rotator cuff tears with a disruption of its anterior tendon (Fig. 1, *B*).^18^^,^^24^ It is hard to know exactly which anatomic structure in the anterior tendon is responsible for the deleterious effects on the SS muscle because the CHL and SS cords are adjacent to each other and partially linked together by interdigitating collagen fibers.^7^^,^^15^^,^^16^^,^^30^ However, the above shoulder abduction force results indicate that the SS cord, and not the CHL, functionally connects the SS muscle to the humerus. When the SS cord is torn, the SS muscle will naturally lose its native tension. Gerber et al have shown that an IS-simulated tear in a sheep model led to irreversible muscle atrophy, infiltration by fat cells, increased interstitial connective tissue, and impairment of physiological properties.^12^

The average anterior-to-posterior humeral footprint widths of the CHL, SS cord, SS strap, and IS tendons starting behind the bicipital groove were 0 mm to 3 mm, 3 mm to 11 mm, 11 mm to 20 mm, and 20 mm to 40 mm, respectively (Table IV). The total anterior-to-posterior width of the CHL footprint was 12 mm, including 9 mm anterior to the posterior bicipital groove (Fig. 6, *B*). Namdari et al found that the SS tears with an intact anterior tendon group started on average 10 ± 4 mm behind the bicipital tendon/groove and progressed 11 ± 4 mm in a posterior direction.^24^ When this study’s footprint widths are compared to the intact anterior tendon footprint widths in Namdari et al, the CHL and SS cord footprints are 100% intact, but the SS strap footprint is 100% torn. In rotator cuff tears with a SS disrupted anterior tendon, the SS tear originated at the bicipital groove and extended 20 ± 7 mm in a posterior direction, indicating rupture of 25% (3 mm/12 mm) of the CHL, 100% (8 mm/8 mm) SS cord, and 100% (9 mm/9 mm) SS strap humeral footprints.^24^ In summary, the SS cord footprints were intact without SS fatty degeneration and torn with SS fatty degeneration. The CHL was largely intact in cases with and without SS fatty degeneration, and the SS strap was torn in both fatty states. The positive relationship between the condition of the SS cord footprint and SS fatty degeneration implies that an intact SS cord plays a role in preventing SS fatty degeneration. The link between the SS cord and SS fatty degeneration is further strengthened by the above mechanical finding that the SS cord transferred a substantial amount of shoulder abduction force to the humerus.

It has been accepted in the orthopedic community that the rotator cable acts as a suspension bridge, transferring the SS and IS muscle force to the humerus and bypassing the crescent area. ^6^^,^^10^^,^^13^^,^^21^^,^^29^ However, the present study’s shoulder abduction force results suggest otherwise. The anterior rotator cable is connected to the greater tuberosity through the CHL.^6^^,^^7^ Releasing the CHL did not decrease the shoulder abduction force (*P* ≥ .081). The role of the rotator cable in SS and IS force transfer is controversial. In a biomechanical study, Halder et al showed that side-to-side repairs of two-third SS tears restored shoulder abduction force to native values, which supports the concept of the rotator cable carry load around the partially released SS tendon.^13^ Mesiha et al reported that rotator cable tears vs. crescent tears had a greater tear gap distance (*P* = .002), tendon stiffness (*P* = .002), and asymmetric regional strain (*P* < .05).^21^ However, other investigators point out that their rotator cable releases may have involved more than the rotator cable’s insertion by also cutting the SS cord.^32^ Wang et al questioned the role of the rotator cable in carrying abduction force to the humerus by reporting that an entire rotator cable release, vs. no release, resulted in no increase in middle deltoid force required for 60° of dynamic shoulder abduction.^35^ Another mechanical study showed that a single or complete release of the rotator cable’s insertions did not change in shoulder abduction force (*P* ≥ .180), principal strain magnitude (*P* ≥ .740), or strain direction (*P* ≥ .493) in either the rotator cable or crescent area.^32^

Surprisingly, the release of the SS strap did not cause a substantial drop in shoulder abduction force (*P* ≥ .264) (Table II). Shoulder abduction force did significantly drop after releasing both the SS strap and IS tendons (*P* < .001). The muscular cross-sectional area of the SS cord is 2.3 times larger than the SS strap (*P* < .001), implying that the SS cord can generate more contractile force than the SS strap.^30^^,^^34^ Other mechanical studies have shown that full, compared to partial, SS release decreases shoulder abduction force.^13^^,^^27^ The SS strap may not carry the full SS cord abduction load through the SS cord-to-SS strap tendinous connection as previously reported.^31^

The anterior-to-posterior distance between the SS cord and SS strap and the SS strap and IS was 11 ± 2 mm and 20 ± 3 mm from the posterior bicipital groove, respectively. A clinical US study reported that the most common location of a partial-thickness rotator cuff tear was 12 mm to 15 mm posterior to the bicipital groove.^18^ The investigators proposed that rotator cuff tears may initiate at the above location and placed the site of rotator cuff tear initiation at the junction between the SS and IS or within the IS tendons based on available humeral footprint studies.^18^ The present study’s anatomic measurements locate the site of rotator tear initiation closer to the junction between the SS cord and SS strap tendons (11 mm) than the interface between the SS and IS tendons (20 mm). The SS cord and SS strap carry different muscle loads with similar tendon cross-sectional areas, which could cause nonuniform strain between the two structures and initiate collagen fiber disruption and subsequent tearing.^30^ It is important to understand the anatomical initiation site of rotator cuff tears to model progression over time.^4^

Scanned cross-section images of the SSc, CHL, SS cord, SS strap, and IS tendons at their midpoint clearly show the above structures are connected to each other but not uniform in their shape or size (Fig. 6, *A*). The mid cross-sectional area of the structures did not correlate to their role in shoulder abduction force transmission. The CHL and SS cord have similar areas of 59 ± 39 mm^2^ and 54 ± 24 mm^2^ (*P* = .546), respectively, but the SS cord transfers abduction force to the humerus, while the CHL plays no role in force transmission. Other investigators have also demonstrated this interconnectivity between the above structures and postulated that the CHL (rotator interval) is important for shoulder range of motion and stability.^7^^,^^15^^,^^16^ Releasing the CHL near the coracoid leads to an increase in external rotation and flexion and a decrease in inferior and posterior humeral stability.^15^^,^^16^ In the present study, releasing the CHL alone or CHL, SS cord, SS strap, and IS did not cause a substantial change in AP or SI humeral apex motion (*P* ≥ .610). However, the present study was not designed to test for the effects of CHL and rotator cuff humeral releases on range of motion and joint stability. The study does show that releasing the anterior superior rotator cuff from its footprint does not destabilize the humerus at 0° and 30° of abduction in the scapular plane.

The insertional footprint shapes and dimensions of the CHL, SS cord, SS strap, and IS are depicted and stated in Figures. 5, *B* and 6, *B* and Table IV. The present study and other investigators have demonstrated that the posterior part of the CHL humeral insertion is sandwiched between the bicipital groove and the SS cord.^5^^,^^7^^,^^16^ The SS cord was inserted at an average of 3 ± 1 mm from the posterior bicipital groove. Other researchers have reported that the SS footprint starts immediately adjacent to the bicipital groove; however, the CHL humeral footprint was not shown in their work.^8^^,^^22^^,^^23^^,^^26^ Their dissection focus was on the rotator cuff and not the CHL.^8^^,^^22^^,^^23^^,^^26^ The distal interval between the CHL and the SS cord is difficult to separate, and part of the CHL footprint could have blended into the SS cord and vice versa.^2^^,^^3^^,^^11^^,^^16^^,^^25^

There are several limitations in the current study. This is a biomechanical study that measured only shoulder abduction force at 0° and 30° without a deltoid force, and it may not fully model the clinical state. However, simplifying the mechanical model can limit confounding variables.^32^ The study simulates a CHL and SS cord rupture by cutting its fibers off their respective footprints, and our model may not measure the effect of shared force transfer between the CHL and SS cord on shoulder abduction force. However, US and magnetic resonance imaging studies of rotator cuff tears typically do not image the status of CHL/SS cord interdigitating fibers, and yet, clinical abduction forces are still valuable preoperative and postoperative measurements. The footprint anatomy was based in part on identifying the structures from the bursal side. Despite this limitation, our average anterior-to-posterior width measurements of the SS (17 mm) and IS (20 mm) footprints are within the ranges reported by other investigators: Curtis et al SS (23 mm) and IS (29 mm), Dugas et al SS (16 mm) and IS (16 mm), Minagawa et al SS (medial 23 mm; lateral 13 mm) and IS (23 mm), and Mochizuki et al SS (medial 13 mm; lateral 1 mm) and IS (medial 20 mm; lateral 33 mm).^8^^,^^9^^,^^22^^,^^23^ The strengths of the study are randomized study design, number of specimens, physiological rotator cuff loading, and noncontact methods to quantify tendon and footprint dimensions.

## Conclusion

The SS cord, and not the CHL, is likely the key structure responsible for the transmission of anterior shoulder abduction force in anterosuperior rotator cuff tears. Based on footprint anatomy, the SS cord is intact in small- to medium-sized rotator cuff tears without SS fatty degeneration. Abduction force transmission results, footprint anatomy, and clinical tear location^24^ points to the SS cord as the key structure preventing fatty muscle degeneration in small- to medium-sized rotator cuff tears. Repairing the SS cord in small- to medium-sized rotator cuff tears could be an essential step in improving abduction strength and preventing SS muscle fatty infiltration.

## Disclaimers

Funding: No external support was given for this project.

Conflicts of interest: The authors, their immediate families, and any research foundation with which they are affiliated have not received any financial payments or other benefits from any commercial entity related to the subject of this article.
