# Supplementary material for: Load transmission via the supraspinatus cord prevents muscle fatty degeneration, a biomechanical study
Source: JSES Int. 2025 Feb 19;9(4):1098–106. doi: 10.1016/j.jseint.2024.12.024 (PMC12435036; doi:10.1016/j.jseint.2024.12.024)
Supplement: Supplementary Methods [file mmc1.docx]

Supplement - Material and Methods

Shoulder Simulator

The shoulder simulator provided load to each tied muscle using 6 servo-driven actuators (Parker Hannifin) fixed to a metal frame (Figure 4).^32^ The 6 lines of muscle pull were replicated by low friction cables passing through pulleys that each connected to a single-DOF (degree-of-freedom) load cell (MLP-100; Transducer Techniques, Inc.) (accuracy ±0.25% RO, non-repeatability = 0.05% RO) to each actuator. The actuators operated using force feedback control (LabVIEW; National Instruments). This allowed for the precise loading of the six distinct rotator cuff tendons. The simulator also allowed for testing at multiple abduction angles using a custom-made arc. Each specimen was positioned within the machine so that the glenohumeral joint was appropriately centered. A 6-DOF load cell (ATI Industrial Automation; Apex) (accuracy ± 0.1 N) measured shoulder abduction force at the unconstrained distal humerus; the force value was recorded by a data acquisition system (National Instruments).

*Mechanical Testing*

The mechanical testing protocol has been previously published.^32^ The humeral head was centered in the glenoid by using live C-arm imaging, while gradually adjusting each of the 6 actuators to 10N. Once the humeral head was centered, a 10N preload was maintained for 5 minutes to eliminate hysteresis. Each specimen underwent cyclic loading from 10N to physiological load with a frequency of 0.25 Hz. The physiological loads (SS Cord 56N, SS Strap 24N, IS 90N, TM 97N, upper SSc 108N, lower SSc 127N) were based on muscle cross-sectional area and electromyographic activity.^17,28,30^ Four complete cycles were measured in force and position, with values recorded at the peak of the 4^th^ cycle. The testing protocol was designed to prevent sustained loading and to reduce hysteresis.

The above testing protocol was repeated for the native case and after each sequential tendon release. The specimens were randomly assigned to either a CHL-First or SS cord-First release group (Excel; Microsoft). CHL-First Group was released in the order of CHL, SS cord, SS strap, and IS. The SS cord-First Group was released in the order of SS cord, CHL, SS strap, and IS. Each specimen was tested at 0° and 30° of abduction in the scapular plane because the SS is the main shoulder abductor.^1,20,33^ The humeral load cell was calibrated to cancel out the effects of increased gravity at 30°.

*Humeral Head Translation*

Humeral head translation was quantified by measuring anterior-posterior (AP) and superior-inferior (SI) humeral apex motion using a motion analysis system (Spicatek; Sica Technology), the details have been previously published.^31^ During mechanical testing, the relative motion between the 4 humeral and 4 scapular markers was tracked using the motion analysis system. After testing, each specimen was dissected down to bone and care was taken to leave the 8 markers undisturbed. Sixteen points around the glenoid perimeter were identified and recorded in relation to the 4 scapular markers using the same motion analysis system. The AP and SI directions relative to the glenoid center were established using the most anterior-posterior and superior-inferior glenoid points, respectively. Similarly, the position of the humeral head apex, the point on the surface of the humeral head at its geometric center, was found with respect to the 4 humeral markers.^19^ Using the recorded relationship between the humeral and scapular markers during each test, the AP and SI humeral apex motion was quantified for all test conditions.

*Ligament/Tendon Thicknesses and Foot Print Location, Size, and Area Measurements*

After mechanical testing, the rotator cuff tendinous capsular complex was sharply separated from the scapula and humerus, leaving the soft tissues (Figure 5A) and the humeral footprints for measurements (Figure 5B). The matching 20 rotator cuff tendinous complexes and humeri were laser scanned using a non-contact optical micrometer (FaroArm, Faro Inc).^32^ Three-dimensional solid replicas of the rotator cuff tendinous complexes and humeri were created from the laser scan data using modeling software (Geomagic; 3D Systems).^32^ The CHL, SS cord, SS strap, and IS thickness (SI), width (AP), and cross-sectional areas were measured half the distance from the medial footprint to the musculotendinous junction (Figure 6A). The CHL, SS cord, SS strap, and IS insertional footprint locations, anterior-to-posterior widths, medial-to-lateral lengths, and areas were quantified from the models (Figure 6B). The 20 anatomic measurements were then averaged and reported to the nearest whole mm. Our laboratory has previously reported the accuracy of the laser micrometer to be 0.01mm.^37^
